# Symmetry Breaking and Cooperative Spin Crossover in a Hofmann-Type Coordination Polymer Based on Negatively Charged {Fe^II^(μ_2_-[M^II^(CN)_4_])_2_}_*n*_^2*n*–^ Layers (M^II^ = Pd, Pt)

**DOI:** 10.1021/acs.inorgchem.3c01332

**Published:** 2023-08-01

**Authors:** Alejandro Orellana-Silla, Manuel Meneses-Sánchez, Rubén Turo-Cortés, M. Carmen Muñoz, Carlos Bartual-Murgui, José Antonio Real

**Affiliations:** † Departamento de Química Inorgánica, Instituto de Ciencia Molecular (ICMol), Universidad de Valencia, Paterna, 46980 Valencia, Spain; ‡ Departamento de Fisica Aplicada, Universitat Politècnica de València, Camino de Vera s/n, 46022 Valencia, Spain

## Abstract

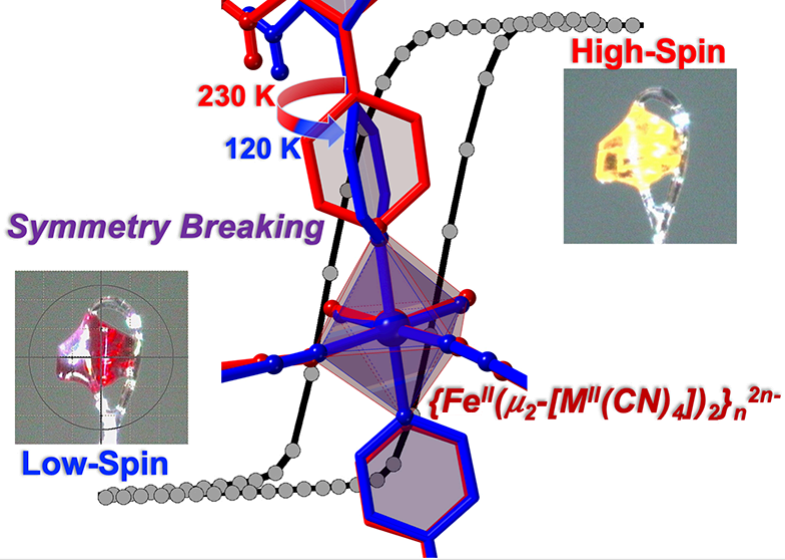

We report herein the synthesis and characterization of two unprecedented isomorphous spin-crossover two-dimensional coordination polymers of the Hofmann-type formulated {Fe^II^(Hdpyan)_2_(μ_2_-[M^II^(CN)_4_])_2_}, with M^II^ = Pd, Pt and Hdpyan is the *in situ* partially protonated form of 2,5-(dipyridin-4-yl)aniline (dpyan). The Fe^II^ is axially coordinated by the pyridine ring attached to the 2-position of the aniline ring, while it is equatorially surrounded by four [M^II^(CN)_4_]^2–^ planar groups acting as *trans* μ_2_-bidentate ligands defining layers, which stack parallel to each other. The other pyridine group of Hdpyan, being protonated, remains peripheral but involved in a strong [M^II^-C≡N···Hpy^+^] hydrogen bond between alternate layers. This provokes a nearly 90° rotation of the plane defined by the [M^II^(CN)_4_]^2–^ groups, with respect to the average plane defined by the layers, forcing the observed uncommon bridging mode and the accumulation of negative charge around each Fe^II^, which is compensated by the axial [Hdpyan]^+^ ligands. According to the magnetic and calorimetric data, both compounds undergo a strong cooperative spin transition featuring a 10–12 K wide hysteresis loop centered at 220 (Pt) and 211 K (Pd) accompanied by large entropy variations, 97.4 (Pt) and 102.9 (Pd) J/K mol. The breaking symmetry involving almost 90° rotation of one of the two coordinated pyridines together with the large unit-cell volume change per Fe^II^ (*ca*. 50 Å^3^), and subsequent release of significantly short interlayer contacts upon the low-spin → high-spin event, accounts for the strong cooperativity.

## Abstract 2

The [M^II^(CN)_4_]^2−^ building blocks of the reported compounds (M^II^ = Pd, Pt) act as *trans* μ_2_-bridging ligands linking the Fe^II^ ions through the four equatorial sites. The accumulated negative charge is canceled by *in situ* protonation of the axial Hdpyan^+^ ligands. This unprecedented 2D Hofmann-type structure, featuring strong intermolecular interactions, provides cooperative hysteretic spin transitions coupled with the loss ↔ gain of an inversion center through the rotation of a pyridine ring.

## Introduction

Octahedral Fe^II^ spin-crossover (SCO) complexes are a type of switchable molecular materials that have attracted much attention because of their potential as essential components in sensors and memory devices.^1,2^ In these molecular materials, the energy gap between the low-spin (LS, t_2g_^6^e_g_^0^) and high-spin (HS, t_2g_^4^e_g_^2^) states is of the order of magnitude of the thermal energy. Consequently, they can be reversibly interconverted by changes in temperature and/or pressure, by light irradiation and even by host–guest interactions. The LS ↔ HS switch is coupled with remarkable changes in the magnetic, calorimetric, optical, and electrical properties of the material. Furthermore, associated with the antibonding character of the e_g_ orbitals, their population–depopulation has important consequences in the size and shape of the SCO centers. Depending on the degree of coupling between the e_g_ ↔ t_2g_ internal electron transfer and the structural changes, the SCO profile may be gradual or abrupt and even with thermal hysteresis (strong cooperativity) but also with steps when the crystal packing favors opposing elastic interactions (elastic frustration) between the SCO centers.^3−14^ In addition, whatever the profile, the SCO event may be coupled with crystallographic phase transitions,^15−26^ which often condition the kinetics and cooperativity of the SCO.^27−29^

**Scheme 1:**
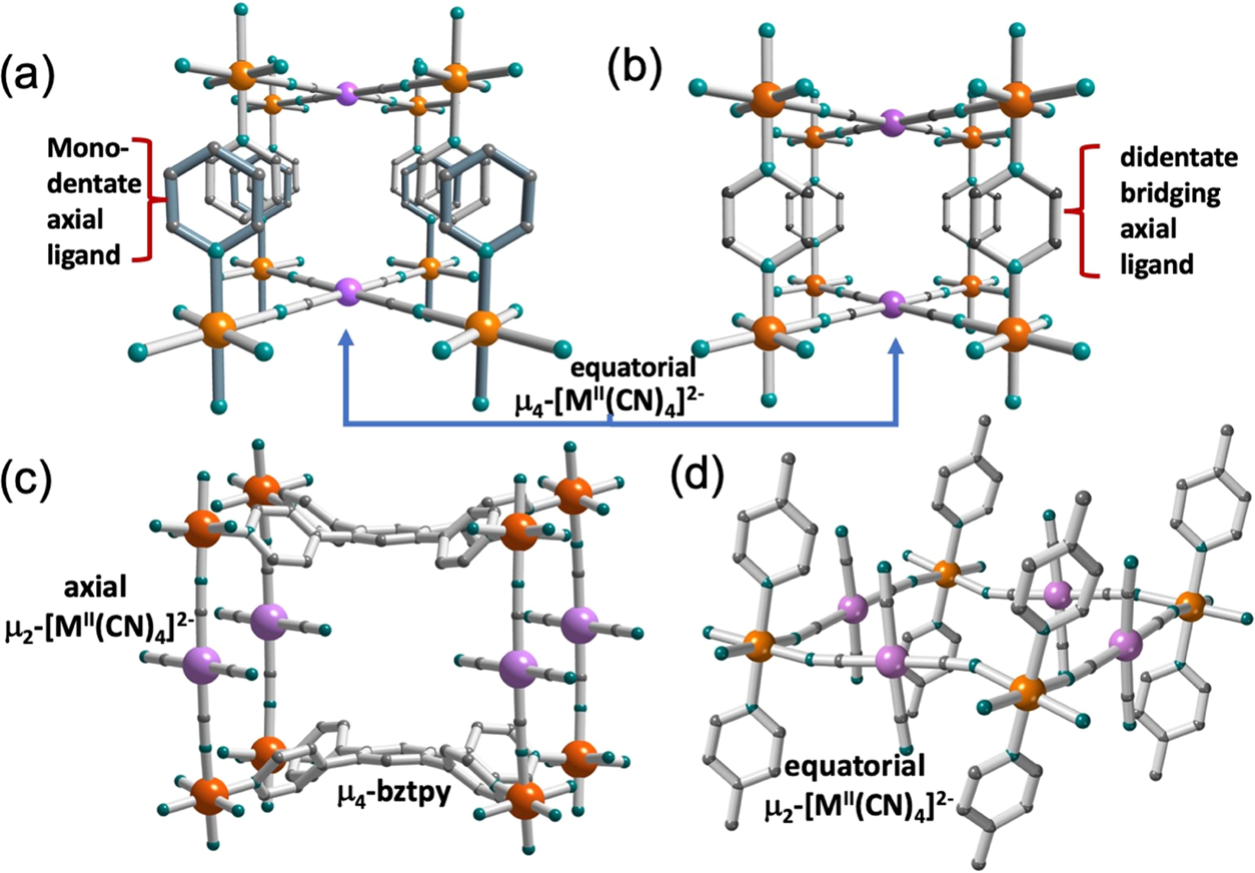
Typical 2D (a) and 3D (b) Hofmann-Type Structures Based on Equatorial [μ_4_-M^II^(CN)_4_]^2–^ Bridging Ligands. Reverse 3D Hofmann-Type Structure Based on the Axial [μ_2_-M^II^(CN)_4_]^2–^ and μ_4_-Tetradentate-Pyridine-Like Equatorial Bridging Ligands (c). Fragment of a Charged {Fe(μ_2_-[M^II^(CN)_4_])_2_}_n_^2n–^ Layer Based on Equatorial [μ_2_-M^II^(CN)_4_]^2–^ Ligands, and (d) (The Protonated Axial Ligand Is Partially Shown)

The last two decades have witnessed the development of a new series of porous and non-porous 2D and three dimensional (3D) Hofmann-type Fe^II^ SCO coordination polymers based on [M^II^(CN)_4_]^2–^ (M^II^ = Ni, Pd, Pt) linkers.^30−32^ These anionic metallo-ligands usually work coordinating the four equatorial positions of the Fe^II^ center acting as tetradentate nodes assembling four Fe^II^ centers, thereby defining dense {Fe μ_4_-[M(CN)_4_]}_n_ grids and imparting electro-neutrality to the polymer. The axial coordination sites of Fe^II^ centers are completed with monodentate (Scheme 1a) or bridging bis-monodentate (Scheme 1b) ligands that contain N-imine donor 5-^33−40^ or 6-^41−58^ membered rings. As far as we know, there are two exceptions to this general rule. Triki and co-workers have shown that the use of a strong chelate bidentate ligand such as quinoline-8-amine can compete with [M^II^(CN)_4_]^2–^ (M^II^ = Ni, Pt) for the equatorial positions of the octahedron to give infinite chains {Fe(aquin)_2_[μ_2_-M(CN)_4_]}_n_, where [M^II^(CN)_4_]^2–^ acts similarly as [M^I^(CN)_2_]^−^ (M^I^ = Ag, Au) ligands do. Both compounds undergo a cooperative SCO with narrow thermal hysteresis (ca. 2 K) centered at 145 (Ni) and 133 K (Pt).^59^ A very different situation has recently been reported by Yao, Tao, and co-workers who assembled Fe^II^, [Pt^II^(CN)_4_]^2–^ and the tetradentate ligand bztpy = 1,2,4,5-tetra(4-pyridyl)benzene. In this case, bztpy is not a strong chelate ligand but a pyridine-type one, which competes against the [Pt^II^(CN)_4_]^2–^ ligand for the four equatorial Fe^II^ positions to afford a stacking of positively charged 2D layers. Contrary to what is usual, these layers are pillared by the [Pt^II^(CN)_4_]^2–^ ligands that are relegated to acting as a *trans* bis-monodentate ligand, thereby affording a new type of 3D SCO porous material so-called “reverse Hofmann-type” formulated {[Fe(μ_4_-bztpy)μ_2_-Pt(CN)_4_]·0.5bztpy·*n*Solvent (Scheme 1c). Most probably, the stabilization of this uncommon framework is favored by metric compatibility of the building blocks and the templating effect of the clathrated molecule of bztpy. This compound undergoes incomplete one- or two-step SCO (depending on the solvent) with very narrow hysteretic SCO behavior accompanied by a drastic color change similar to that shown by the title compound.^60^

Herein, we report the synthesis and characterization of {Fe^II^(Hdpyan)_2_(μ_2_-[M^II^(CN)_4_])_2_}, an unprecedented 2D Hofmann-type SCO framework stemming from assembling Fe^II^, the bis-monodentate ligand 2,5-(dipyridin-4-yl)aniline (dpyan), and [M^II^(CN)_4_]^2–^ (M^II^ = Pd, **dpyanPd**; Pt **dpyanPt**), which represents a third exception to the aforementioned general rule. *In situ* protonation of one pyridine ring of the axial dpyan ligands favors the formation of negatively charged {Fe(μ_2_-[M^II^(CN)_4_])_2_}_n_^2n-^ layers mutually reaching electro-neutrality (Scheme 1d). The resulting Pd and Pt isomorphous compounds undergo strong cooperative spin crossover coupled with symmetry breaking.

## Results

### Synthesis

Both compounds, **dpyanM** (M = Pt, Pd), were prepared exclusively as single crystals using the layering liquid-to-liquid slow diffusion method in test tubes (see Experimental Section). The homogeneity of the crystalline bulk samples (15–40 mg) was checked comparing their corresponding powder X-ray patterns (see Figure S1) with the calculated ones derived from single-crystal analysis (*vide infra*) as well as by elemental analysis. The IR spectrum of both derivatives, in addition to the more or less modified characteristic modes of the dpyan ligand, is characterized by the presence of two asymmetric peaks in the wavenumber window of the C≡N stretching vibrational mode of the [M^II^(CN)_4_]^2–^anions [ν_Pd_ = 2142 (m), 2169 (s) cm^–1^; ν_Pt_ = 2139 (m), 2162 (s) cm^–1^] (see Figure S2).

### Spin-Crossover Behavior

#### Magnetic Measurements

**Figure 1:**
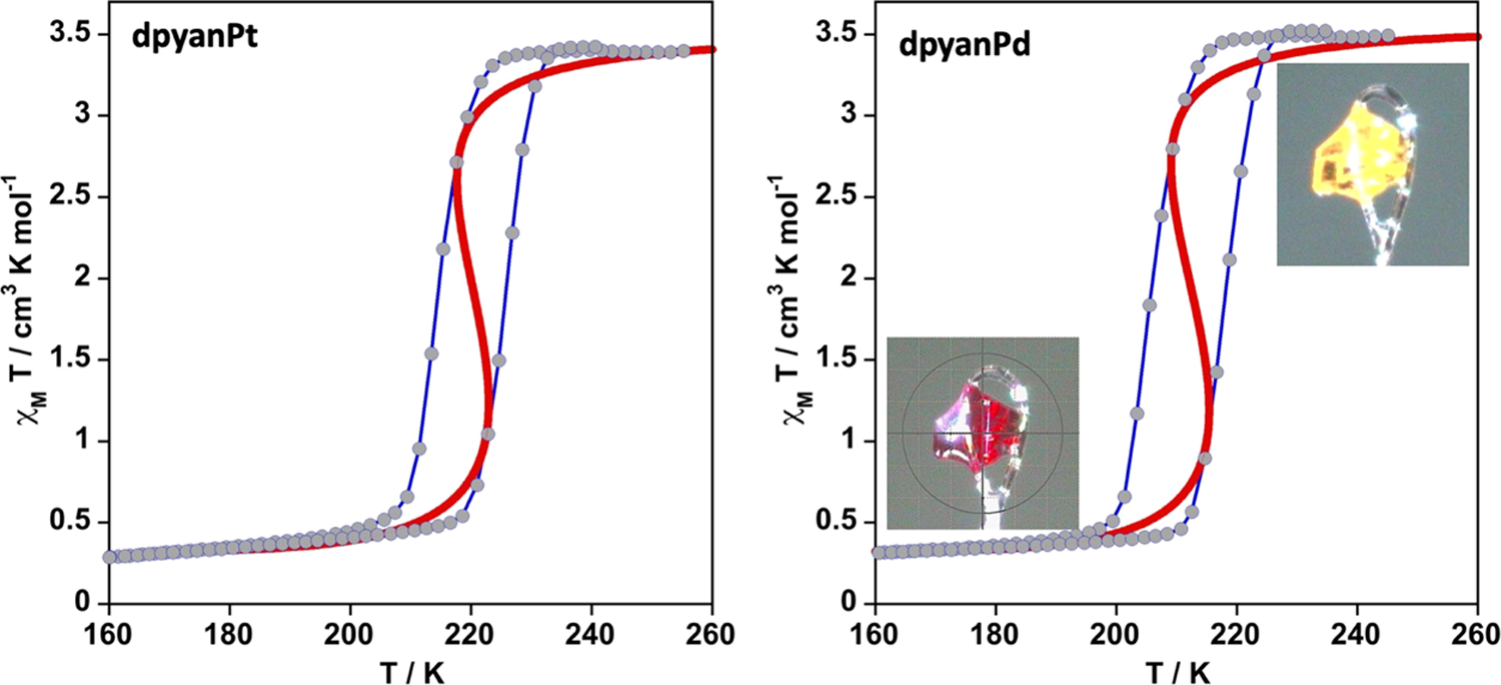
χ_M_*T* versus *T* plots for **dpyanPt** and **dpyanPd**. Solid red line corresponds to simulation of the SCO based on the regular solutions model, see text. Blue lines are for guiding the eyes.

Figure 1 displays the magnetic properties of both compounds expressed as the thermal dependence of the χ_M_*T* product recorded at a temperature rate of 2 K min^–1^, being χ_M_ the molar magnetic susceptibility and *T* the temperature (see the Experimental Section for more details). At 250 K, the χ_M_*T* value is in the interval 3.45–3.53 cm^3^ K mol^–1^, for both compounds, indicating that the Fe^II^ centers are in the paramagnetic HS state. Upon cooling, χ_M_*T* remains practically constant down to 230 K for **dpyanPt** and 220 K for **dpyanPd**. Then, χ_M_*T* drops drastically and reaches a value of ca. 0.32 cm^3^ K mol^–1^ at temperatures lower than 190 K. This χ_M_*T* value is consistent with a substantial transformation of the Fe^II^ centers from the HS state to the LS state, remaining around 10% of the Fe^II^ centers in the HS. The SCO temperatures in the cooling mode, *T*_SCO_^↓^, estimated from the maximum of [∂(χ_M_*T*)/∂*T*] vs *T*, are 215 and 205 K for the Pt and Pd derivatives, respectively. In the heating mode, the χ_M_*T* values do not match those of the cooling mode being the corresponding *T*_SCO_^↑^ values 225 K (Pt) and 217 K (Pd), thus defining a thermal hysteresis Δ*T* = *T*_SCO_^↓^- *T*_SCO_^↑^ ≈ 10–12 K wide, evidencing the occurrence of a cooperative thermal-induced HS ↔ LS transition.

Both derivatives lack light-induced excited spin-state trapping effect (LIESST).^61^ As it is usual for this family of Fe^II^ compounds, their SCO transition takes place accompanied by a drastic reversible change of color from light-yellow in the HS state to deep-red in the LS state (see inset in Figure 1). Taking into account the pseudo-octahedral nature of the [Fe^II^N_6_] chromophores, this fact reflects the change from the ^5^T_2_ → ^5^E medium-weak electronic HS absorption, typically centered at 800–900 nm, to the intense ^1^A_1_ → ^1^T_1_ and ^1^T_2_ absorption LS bands, usually found in the 450–600 nm energy window.^62^

#### Differential Scanning Calorimetry

**Figure 2:**
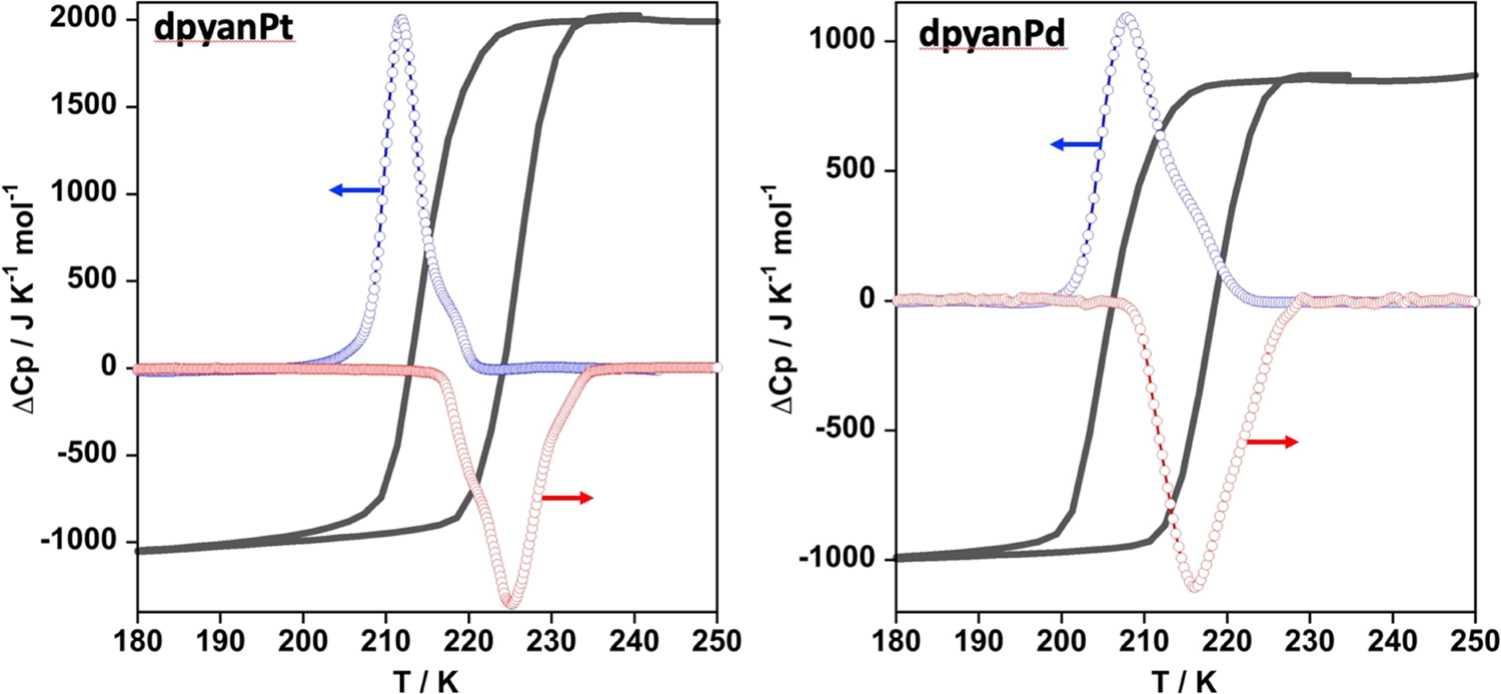
Δ*C*_p_ versus *T* plots for **dpyanM** (M = Pt, Pd). Blue and red circles correspond to the cooling and heating modes. The magnetic curves are shown as black lines. Blue and red lines are for guiding the eyes.

The SCO behavior was also investigated through the thermal dependence of the heat capacity at constant pressure, Δ*C*_p_, obtained from differential scanning calorimetry (DSC) measurements for **dpyanM** (M = Pt, Pd) (Figure 2) (temperature scan rate 10 K/min). The enthalpy values, Δ*H*, for the cooling and heating modes were obtained from integrating the corresponding anomalous Δ*C*_p_ vs *T* plots in the SCO temperature window. The associated entropy values, Δ*S*, were obtained as Δ*H*/*T*_SCO_^DSC^, being *T*_SCO_^DSC^ the temperature of the maximum (cooling) or minimum (heating) of the Δ*C*_p_ vs *T* plot. The *T*_SCO_^DSC^ values obtained from the calorimetric measurements (*T*_SCO_^DSC↓^ = 212.0 and 208.0 K and *T*_SCO_^DSC↑^ = 225.1 and 216.0 K for **dpyanPt** and **dpyanPd**, respectively) agree reasonably well with the corresponding *T*_SCO_^↓^ and *T*_SCO_^↑^ obtained from the χ_M_*T* vs *T* plots. The resulting average variations, Δ*H*^av^, Δ*S*^av^, and *T*_SCO_^DSC^ values are, respectively, 21.3 kJ/mol, 97.4 J/K mol, and 218.5 K for **dpyanPt** and 21.8 kJ/mol, 102.9 J/K mol, and 212 K for **dpyanPd**. The Δ*H* and Δ*S* values found for both compounds are consistent with those usually obtained for Fe^II^ SCO compounds^63^ and, in particular, with Hofmann-type coordination polymers exhibiting strong cooperative SCO behaviors.^64−66^

Simulation of the spin transitions has been carried out using eq 1, derived from the regular solution model^67^
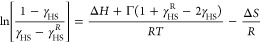
1where Γ is a parameter accounting for the cooperative nature of the spin transition, γ_HS_, the molar HS fraction, is obtained from the magnetic susceptibility through eq 2
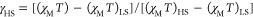
2and γ_HS_^R^, the residual molar fraction, accounting for the HS species blocked at low temperatures, is calculated as follows (eq 3)
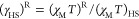
3being (χ_M_*T*), (χ_M_*T*)_HS_, (χ_M_*T*)_LS_ ≈ 0 and (χ_M_*T*)^R^ the value of χ_M_*T* at any temperature of the HS state, of the LS, and the residual HS species blocked at low temperature, respectively. Given that Δ*H*^av^, Δ*S*^av^, *T*_SCO_^DSC^, (χ_M_*T*)_HS_ and (χ_M_*T*)^R^ have been estimated directly from the χ_M_*T* and DSC versus *T* plots, it has been possible to quantify the magnitude of the parameter Γ as ca. 5 kJ/mol for both derivatives, thereby obtaining reasonably good simulation of the spin transition for both compounds (see red solid lines in Figure 1).

### Crystal Structure

**Figure 3:**
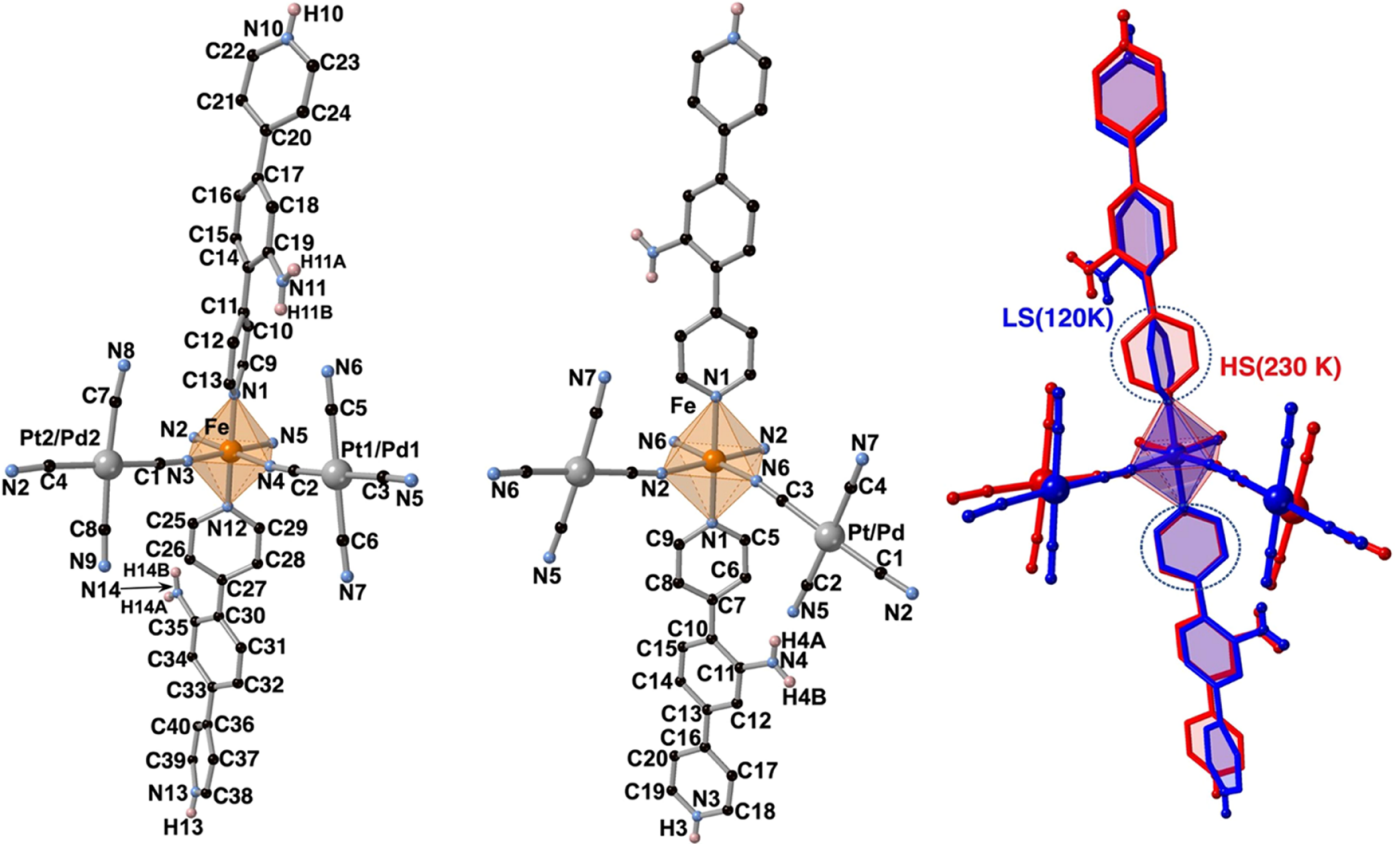
Coordination environment in the LS (left) and HS (middle) state and superposition of both states (right) for **dpyanPt** (red and blue color corresponds to HS and LS, respectively). Blue circles emphasize the relative orientation of the coordinated pyridine rings upon SCO.

The crystal structures have been investigated in the LS (120 K) and HS (230 K) states. Relevant crystallographic data are gathered in Table S1. Both compounds are isomorphous and display the monoclinic *P*2_1_/*n* space group in the HS state. However, they lose the inversion center changing to the monoclinic *Pn* space group in the LS state. Table 1 contains selected bond lengths and angles involving the Fe^II^ coordination environment for **dpyanM** (M = Pt, Pd). The coordination environment in the LS and HS states for the Pt^II^ derivative together with the corresponding atom numbering is shown in Figure 3. The Fe^II^ centers define [FeN_6_] octahedral sites where the equatorial coordination positions are saturated with four [M^II^(CN)_4_]^2–^ (M^II^ = Pd, Pt) anions while the two axial positions are occupied by the organic ligand dpyan. The average <Fe–N> bond length is practically the same for both compounds and change from 1.961/1.958 Å at 120 K to 2.169/2.170 Å at 230 K for M = Pd/Pt, values consistent with the LS and the HS states, respectively, in perfect agreement with the magnetic and calorimetric data. The change in Δ<Fe–N>^HS-LS^ = 0.21 Å is also consistent with the occurrence of a complete SCO behavior which, in addition, is responsible for a change in the unit cell volume, per Fe^II^ center, of 50.85/50.20 Å^3^ for Pd/Pt, respectively.

**Table 1:** Selection of Metal-to-Ligand Bond Lengths (Å) and Angles (°) for **dpyanM** (M = Pd, Pt)

	dpyanPd	dpyanPt		dpyanPd	dpyanPt
	*T* = 120 K			*T* = 230 K	
Fe–N(1)	2.002(11)	2.014(12)	Fe–N(1)	2.236(5)	2.235(5)
Fe–N(2)	1.930(11)	1.925(10)	Fe–N(2)	2.132(5)	2.141(6)
Fe–N(3)	1.928(11)	1.925(10)	Fe–N(6)	2.140(5)	2.135(6)
Fe–N(4)	1.934(10)	1.942(10)			
Fe–N(5)	1.962(11)	1.942(11)			
Fe–N(12)	2.012(11)	1.999(12)			
<Fe–N>	1.961(11)	1.958(12)	<Fe–N>	2.169(5)	2.170(6)
N(1)–Fe–N(2)	89.2(5)	89.8(4)	N(1)–Fe–N(2)	90.2(2)	90.2(2)
N(1)–Fe–N(3)	91.0(5)	91.5(4)	N(1)–Fe–N(6)	91.0(2)	91.2(2)
N(1)–Fe–N(4)	91.3(5)	91.0(4)	N(2)–Fe–N(6)	91.7(2)	92.3(3)
N(1)–Fe–N(5)	89.5(5)	88.4(4)			
N(1)–Fe–N(12)	178.9(6)	179.1(6)			
N(2)–Fe–N(3)	90.8(5)	89.9(5)			
N(2)–Fe–N(4)	177.4(6)	178.8(6)			
N(2)–Fe–N(5)	88.7(4)	89.5(4)			
N(2)–Fe–N(12)	89.7(5)	90.0(5)			
N(3)–Fe–N(4)	91.7(4)	91.0(4)			
N(3)–Fe–N(5)	179.3(6)	179.4(6)			
N(3)–Fe–N(12)	88.6(5)	89.3(5)			
N(4)–Fe–N(5)	88.7(4)	89.5(4)			
N(4)–Fe–N(12)	89.8(5)	89.3(4)			
N(5)–Fe–N(12)	90.8(5)	90.8(5)			
Σ	11.4	8.6	Σ	11.6	14.8

**Figure 4:**
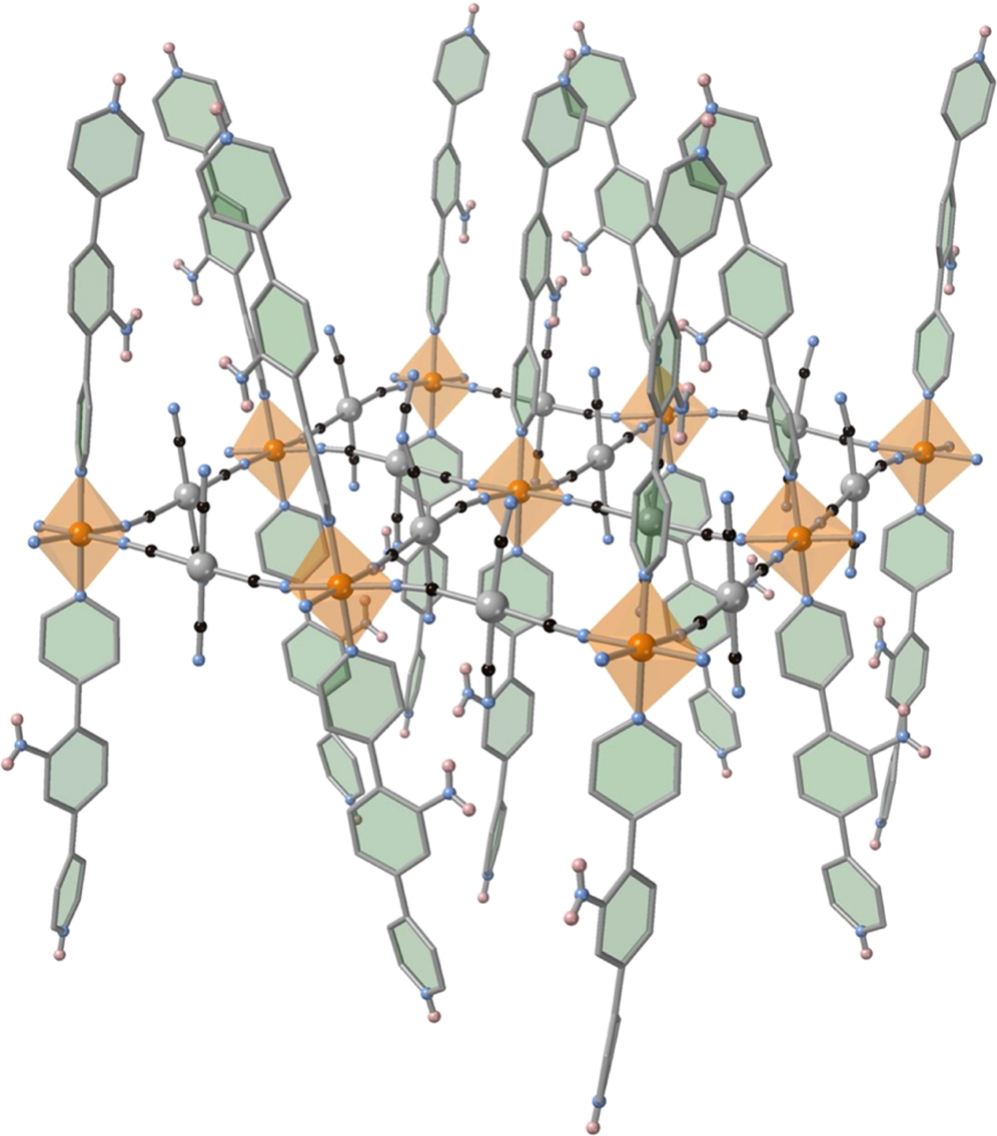
Perspective view of a layer fragment of **dpyanPt** at 120 K.

Interestingly, in the present case, the [M^II^(CN)_4_]^2–^ bridging counterions do not act as square-planar nodes connecting four Fe^II^ centers, thereby generating neutral bimetallic {Fe^II^μ_4_-[M^II^(CN)_4_]}_n_ layers, but as bis-monodentate rod-like ligands using only two *trans* CN groups defining negatively charged {Fe^II^μ_2_-[M^II^(CN)_4_]_2_}^2–^*_n_* layers. The two remaining uncoordinated CN groups, and hence the square-planar [M(CN)_4_]^2–^ groups, are oriented perpendicularly to the average plane defined by the layers (see Figure 4). The layers are not perfectly planar since the angles defined by the M(C)–N–Fe connections differ from 180° in the range 6–12° at 120 K increasing significantly up to 12–21° at 230 K, making the layers more corrugated in the HS state. Alternatively, this corrugation can be described as generated by the separation of the equatorial [FeN_4_]_eq_ planes from the average plane defined by M–Fe layers, which changes from 7.61° (Pd)/7.10° (Pt) at 120 K to 11.65°(Pd)/11.73°(Pt) at 230 K. Obviously, this is reflected in similar changes in the inclination angle of the dpyan ligands.

Fortuitous protonation of the N10 and N13 (120 K) and N3 (230 K) atoms of the dpyan ligand (see Figure 3) provides the required electro-neutrality to the layers. The dpyan ligands adopt a very similar conformation in both derivatives, but the mutual angle defined between the aromatic rings differs significantly on the spin state. More precisely, the angle defined between the rings PhN14H_2_/Py(N13H13)^+^, Py(N12)/Py(N13H13)^+^, and Py(N1)/Py(N12) found, respectively, at 120 K in the range 28–31, 75–76, and 78–79° change to 7.0–8.2, 37.8–41.0, and 0.0° at 230 K, where the equivalent angles are, respectively, PhN(4)H_2_/Py(N3H3)^+^, Py(N1)/Py(N3H3)^+^, and Py(N1)/Py(N1)′ (see Figure 3 right and Table S2).

**Figure 5:**
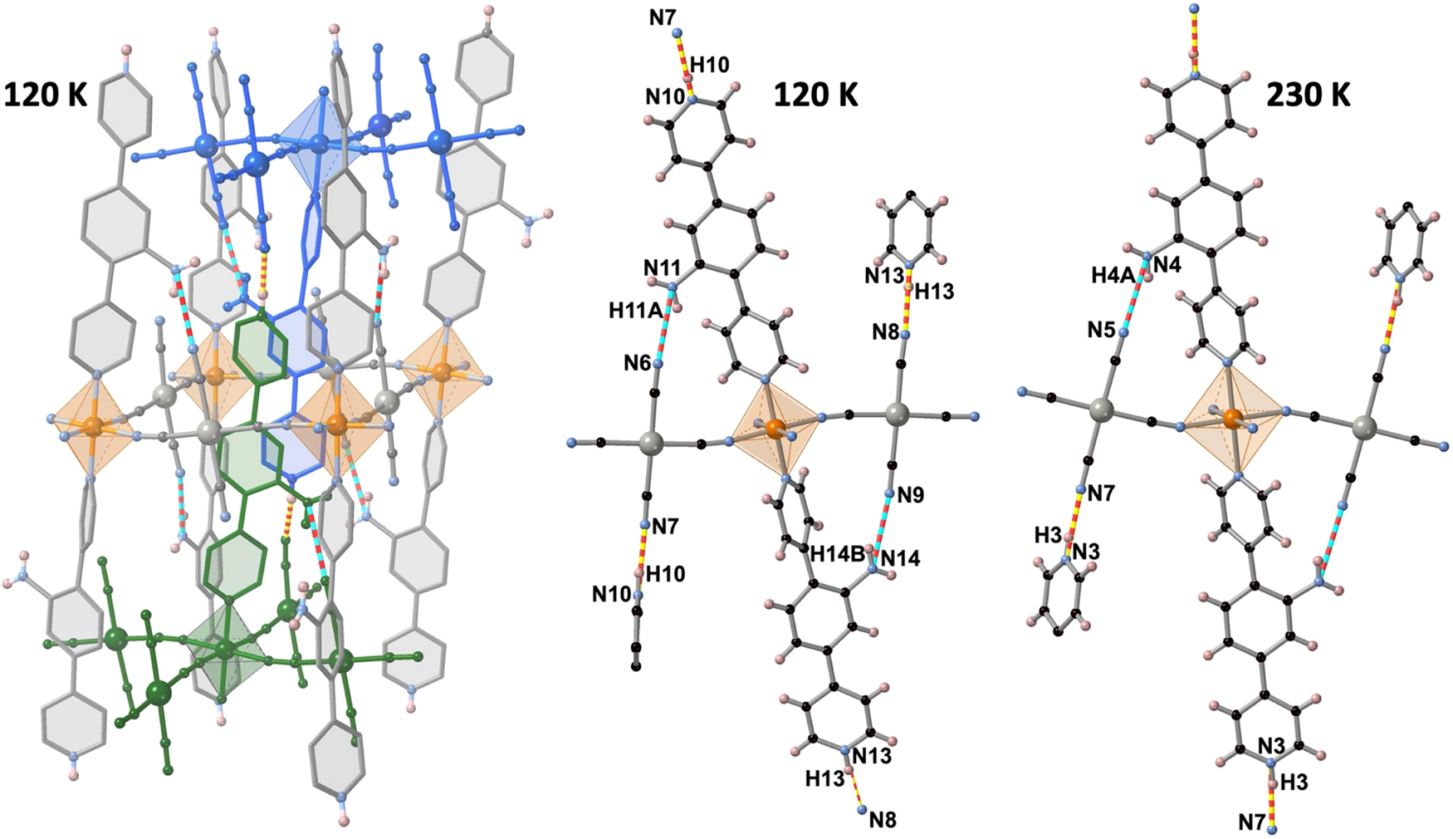
Intra- and inter-layer hydrogen bond interactions for **dpyanM** (M = Pd, Pt) at 120 K (left). Detailed structure fragments showing these interactions at 120 K (middle) and 230 K (right). Red-blue and red-yellow rods represent the intra- and interlayer interactions, respectively.

The layers stack one on top each other in such a way that the dpyan ligands of the *n* + 1 and n – 1 layers penetrate the {Fe_4_[M(CN)_4_]_4_} windows of the layer n (see Figure 5). The singular structure of this coordination polymer is stabilized by a 3D network of intra- and interlayer hydrogen bond interactions. The intralayer interactions are weak and involve the NH_2_ group of the aniline ring and the N atom of one uncoordinated CN group belonging to the [M(CN)_4_]^2-^: d(N6···N11) = 3.056/3.093 Å and d(N9···N14) = 3.098/3.055 Å at 120 K and d(N5···N4) = 3.059/3.064 Å at 230 K for M(Pt/Pd). The interlayer hydrogen bond interactions are strong and involve the protonated pyridine rings, e.g., of the *n* + 1 layer, and the remaining uncoordinated CN group of the layer *n* – 1: d(N8···N13) = 2.747/2.768 Å and d(N7···N10) = 2.754/2.734 Å at 120 K and d(N7···N3) = 2.732/2.745 Å at 230 K for M(Pt/Pd) (see Figure 5).

In addition to the mentioned hydrogen bonds, the interpenetration of two dpyan ligands (marked in green and blue in Figure 5) per {Fe_4_[M(CN)_4_]_4_} window favors the occurrence of a large number of interlayer C···C interactions at 120 K, which involve, on one hand, the terminal CN groups and the aromatic rings of three consecutive layers with d(C8···C37/C8···C38/C2···C24/C3···C16) = 3.241 (3.270)/3.305 (3.308)/3.362 (3.334)/3.361 (3.350) Å for M = Pd (Pt) being the shortest contacts (see Figure S3). On the other hand, the aromatic rings of the *n* + 1 and *n* – 1 layers display an important number of face-to-face π–π interactions (see Figure S4). At 230 K, only one very short contact involving the CN groups and the aromatic rings persists, d(C2···C20) = 3.321/3.346 Å for Pt/Pd, while all C···C distances defining the π–π interactions become larger than 3.6 Å.

## Discussion and Conclusions

The selection of the bis-monodentate dpyan ligand was motivated by two reasons, on one hand, being a long rod-like pillaring building block, it should *a priori* connect, through the axially coordinating sites of the Fe^II^, consecutive parallel stacked {Fe^II^[M^II^(CN)_4_]}_n_ heterometallic layers, thereby generating a new 3D Hofmann-type porous coordination polymer with enhanced porosity. On the other hand, the presence of the amino function introduces in the pores a reactive center that should favor the affinity for specific guest molecules. Surprisingly, an unprecedented exception to the usually expected has been observed since the synthetic procedure described favors the formation of an uncommon 2D layered structure due to *in situ* protonation of the N-pyridine atom attached to the 5-position of the central aniline ring to give (Hdpyan)^+^. The Fe^II^ centers being equatorially surrounded by four [M^II^(CN)_4_]^2–^ anions comply with what it is structurally expected for this family of compounds but with the notable exception that the [M^II^(CN)_4_]^2–^ anionic squares are perpendicularly oriented to the average plane defined by the Fe^II^ and M^II^ atoms. Consequently, the [M^II^(CN)_4_]^2–^ anions act as linear bis-monodentate bridges defining {Fe μ_2_-[M(CN)_4_]}_n_ grids featuring wide {Fe_4_[μ_2_-M(CN)_4_]_4_}_n_ square windows accumulating an excess of two negative charges around each Fe^II^. This negative charge is compensated by the two protonated (Hdpyan)^+^ ligands axially coordinated to the Fe^II^ centers through the pyridine attached to the 2-position of the central aniline ring. Most likely, this unusual coordination mode of the tetracyanometallate ligands, as well as the protonation of the “terminal” pyridine, is the result of a spontaneous concerted process in which minimization of the void space affords a stable framework. Indeed, the NH_2_ group attached to the 1-position of the central aniline ring interact, within the same 2D layer, via weak hydrogen bonding with one of the two terminal N atoms of the [M^II^(CN)_4_]^2–^ anion, while the other one is engaged in a very strong hydrogen bond with the peripheral protonated pyridine of alternated layers, which penetrate the {Fe_4_[μ_2_-M(CN)_4_]_4_}_n_ square windows of the middle 2D layer, thereby conferring an interpenetrated 3D supramolecular nature to the framework where no free void space is available. The presence of two different cyanide groups, namely, one acting as a bridge between M^II^ and Fe^II^ and other terminal involved in an H-bonding network, is reflected on the IR spectra of both derivatives (M^II^ = Pd, Pt) as two asymmetric stretching vibrational modes (see Figure S2).

The two isomorphous compounds undergo a hysteretic spin transition accompanied by symmetry-breaking involving the loss, in the LS state, of the inversion center located at the Fe^II^ center. More precisely, it takes place by rotation of one of the two pyridine rings coordinated to the Fe^II^ from parallel (0°) in the HS state to almost orthogonal (ca. 79°) in the LS state (see Table S2). The HS → LS transition also involves remarkable changes in the relative orientation of the central aniline and protonated pyridine rings. Most likely, this mechanism is driven by the significant change of unit-cell volume (ca. 50 Å^3^ per Fe^II^) and the necessary minimization of the steric hindrance in the congested {Fe_4_[M(CN)_4_]_4_} windows but more particularly between the pyridine (N1–C5–H5 and N1–C9–H9 moieties) and the Fe–N2–C1 equatorial bonds as a consequence of the axial contraction, 0.23 Å, upon SCO. This steric hindrance stems from the particular orientation of both pyridines, which lay close to the vertical of the Fe–N2–C1 equatorial bonds and define relatively small C9–N1–Fe–N2 and C5–N1–Fe–N2 torsion angles (in the range 20–28°). A consequence related to the observed structural changes is the self-grinding of the crystals after several LS ↔ HS cycles ending up as microcrystalline powders without affecting the SCO properties. Furthermore, the concerted cooperative spin transition and symmetry breaking events justify the large values of Δ*S* obtained from DSC measurements. Besides, the occurrence of significant short intermolecular contacts favored by the interpenetrating nature of the structure explains the Γ value, being much larger than 2RT_SCO_, and the aperture of the thermal hysteresis loop. Furthermore, the relatively high *T*_SCO_ values justify the lack of LIESST effect,^68−70^ a similar situation has been recently observed for the 2D {Fe^II^(pyS_2_Me)_2_[Pt^II^(CN)_4_]}_n_.^57^

In summary, here we have described the synthesis and characterization of two isomorphous 2D Hofmann-type SCO coordination polymers featuring a rare μ_2_-coordination mode of the metallo-ligand [M^II^(CN)_4_]^2–^ (M^II^ = Pd, Pt). This fact seems to be correlated with the *in situ* half-protonation of the axial dpyan ligand and the formation of very strong hydrogen bonds between alternate layers forcing the out-of-plane reorientation of the [M^II^(CN)_4_]^2–^ building blocks and generating two interpenetrated supramolecular 3D frameworks. The resulting frameworks characterized by a large number of short contacts exhibit strong cooperative SCO properties.

## Experimental Section

### Materials

Iron(II) tetrafluoroborate hexahydrate, potassium tetracyanoplatinate(II) trihydrate potassium tetracyanopalladate(II) hydrate, and n-tetrabuthylammonium bromide were obtained from commercial sources and used as received without further purification. Tetra-n-butylammonium tetracyanoplatinate(II), tetra-n-butylammonium tetracyanopalladate(II) and the ligand 2,5-di(pyridin-4-yl)aniline (dpyan) were synthesized according to methods described in the literature.^71,72^

### Synthesis of Complexes

#### Synthesis of Fe(dpyan)_2_[M(CN)_4_]_2_ [M = Pt (**dpyanPt**), Pd (**dpyanPd**)]

The samples, exclusively constituted of single crystals, were obtained through a layering liquid-to-liquid slow diffusion method using test tubes. The effective configuration of the layers was as follows: the bottom layer consisted in a containing a mixture of Fe(BF_4_)_2_·6H_2_O (33.7 mg, 0.1 mmol) and dpyan (24.7 mg, 0.1 mmol) previously dissolved, respectively, in 2 mL of H_2_O and 2 mL of MeOH, while the top layer contained a MeOH solution of (n-TBA)_2_[M(CN)_4_] (M = Pt^II^/Pd^II^) (78.4/69.5 mg, 0.1 mmol, 1 mL). Both layers were separated by a 4 mL MeOH:H_2_O (1:1) interphase. The tube was sealed and left to stand at room temperature. Light-yellow cubic single crystals of **dpyanPt** and **dpyanPd** were obtained after 2 weeks (yield: 25–30%). Elemental Analysis: Calculated for **dpyanPt** [C_40_H_28_N_14_FePt_2_ (%)]: C 41.75; H 2.45; N 17.04. Found (%): C 41.23; H 2.50; N 16.89. Calculated for **dpyanPd** [C_40_H_28_N_14_FePd_2_ (%)]: C 49.35; H 2.90; N 20.14. Found (%): C 48.96; H 2.83; N 19.75.

### Physical Characterization

#### Magnetic Measurements

Magnetic measurements were performed on crystalline samples (20–40 mg) with a Quantum Design MPMS-XL-5 SQUID magnetometer working in the 2–400 K temperature range (temperature scan rate 2 K min^–1^) with an applied magnetic field 1 *T*. Experimental susceptibilities were corrected for diamagnetism of the constituent atoms by the use of Pascal’s constants.

#### Calorimetric Measurements

Calorimetric measurements were performed using a differential scanning calorimeter Mettler Toledo DSC 821e. Low temperatures were obtained with an aluminum block attached to the sample holder, refrigerated with a flow of liquid nitrogen gas to avoid water condensation. The measurements were carried out using around 15 mg of crystalline samples sealed in aluminum pans with a mechanical crimp. Temperature and heat flow calibrations were made with standard samples of indium by using its melting transition (429.6 K, 28.45 J g^–1^). An overall accuracy of ±0.2 K in temperature and ±2% in the heat capacity is estimated. The uncertainty increases for the determination of the anomalous enthalpy and entropy due to the subtraction of an unknown baseline.

#### Single-Crystal X-ray Measurements

Single crystals were mounted on a glass fiber using a viscous hydrocarbon oil to coat the crystal and then transferred directly to the cold nitrogen stream for data collection. X-ray data were collected on a Supernova diffractometer equipped with a graphite monochromated Enhance (Mo) X-ray Source (λ = 0.71073 Å). The program CrysAlisPro, Oxford Diffraction Ltd., was used for unit cell determinations and data reduction. Empirical absorption correction was performed using spherical harmonics, implemented in the SCALE3 ABSPACK scaling algorithm. The structures were solved by direct methods using SHELXS-2014 and refined by full matrix least-squares on *F*^2^ using SHELXL-2014.^73^ Non-hydrogen atoms were refined anisotropically, and hydrogen atoms were placed in calculated positions refined using idealized geometries (riding model) and assigned fixed isotropic displacement parameters. CCDC files, 2254120–2254123, contain the supplementary crystallographic data for this paper. These data can be obtained free of charge from The Cambridge Crystallographic Data Center via www.ccdc.cam.ac.uk/data_request/cif.

#### Infrared Spectra

The solid-state absorption IR spectrum was recorded with an Agilent Technologies Cary 630-FTIR spectrometer equipped with a diamond micro-ATR accessory in the 4000–400 cm^–1^ range.

#### Elemental Analyses

(C, H, N) were performed with a CE Instruments EA 1110 CHNS Elemental analyzer.

#### Powder X-ray Diffraction

Powder X-ray diffraction measurements were performed on a PANalytical Empyrean X-ray powder diffractometer (monochromatic Cu Kα radiation) in a capillary measurement mode.

## Supporting Information Available

Details of the crystal data collection and refinement parameters as well as relative angles defined between the different aromatic rings (Tables S1 and S2); experimental and calculated X-ray diffraction patterns (Figure S1); room-temperature infrared spectra (Figure S2); short intermolecular contacts at 120 K (Figures S3 and S4) (PDF)
