# Supplementary material for: Symmetry Breaking and Cooperative Spin Crossover in a Hofmann-Type Coordination Polymer Based on Negatively Charged {FeII(μ2-[MII(CN)4])2}n2n– Layers (MII = Pd, Pt)
Source: Inorg Chem. 2023 Aug 1;62(32):12783–92. doi: 10.1021/acs.inorgchem.3c01332 (PMC10428219; doi:10.1021/acs.inorgchem.3c01332)

Supporting Information

Symmetry breaking and cooperative spin crossover in a Hofmann-type coordination polymer based on negatively charged $\{Fe^{II}(\mu_2\text{-}[M^{II}(CN)_4]_2\}_n^{2n-}$ layers (M^{II} = Pd, Pt).

Alejandro Orellana-Silla, Manuel Meneses-Sánchez, Rubén Turo-Cortés, M. Carmen Muñoz, Carlos Bartual-Murgui and José Antonio Real

Contents

- Experimental and calculated X-ray diffraction patterns for **dpyanM** (**Figure S1**). ***Page 2***
- Room-temperature Infra-red spectrum of **dpyanM** (**Figure S2**). ***Page 2***
- Selected crystallographic data for **dpyanM** (M = Pd, Pt). (**Table S1**). ***Page 3***
- Relative angles defined between the different aromatic rings for **dpyanM** (**Table S2**). ***Page 3***
- Short intermolecular contacts at 120 K for **dpyanM** (M = Pd, Pt) (**Figure S3**). ***Page 4***
- Short intermolecular π-π contacts at 120 K for **dpyanM** (M = Pd, Pt) (**Figure S4**). ***Page 5***

Figure S1. Experimental (blue line) and calculated (red line) X-ray diffraction patterns for **dpyanPt** and **dpyanPd**. Experimental patterns were recorded at 295 K while the simulated ones correspond to the single crystal data recorded at 230 K, both in the HS state.

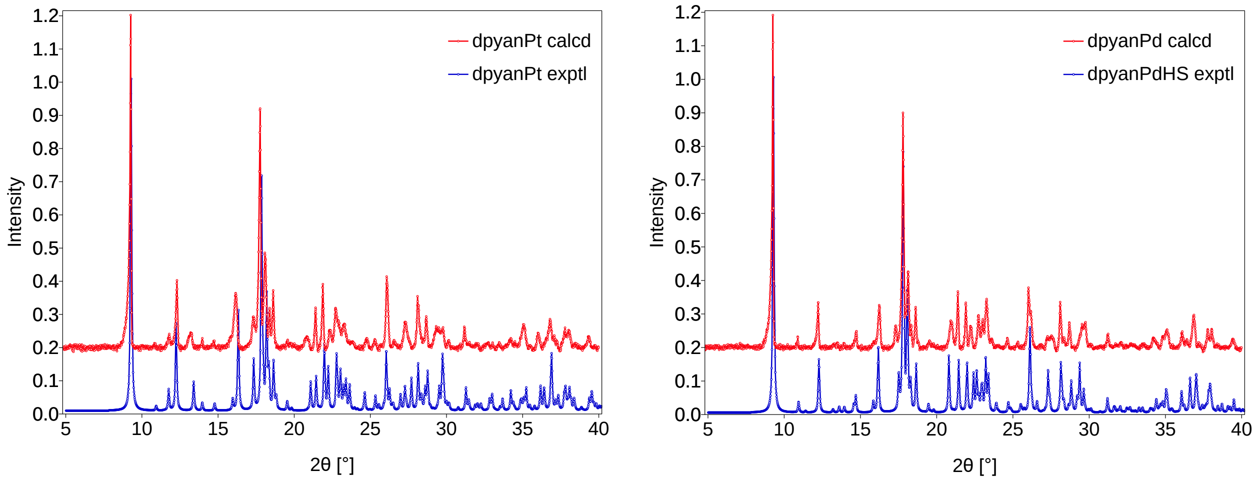

Figure S2. Room temperature Infra-red spectrum of **dpyanPt** (black line) and **dpyanPd** (red line).

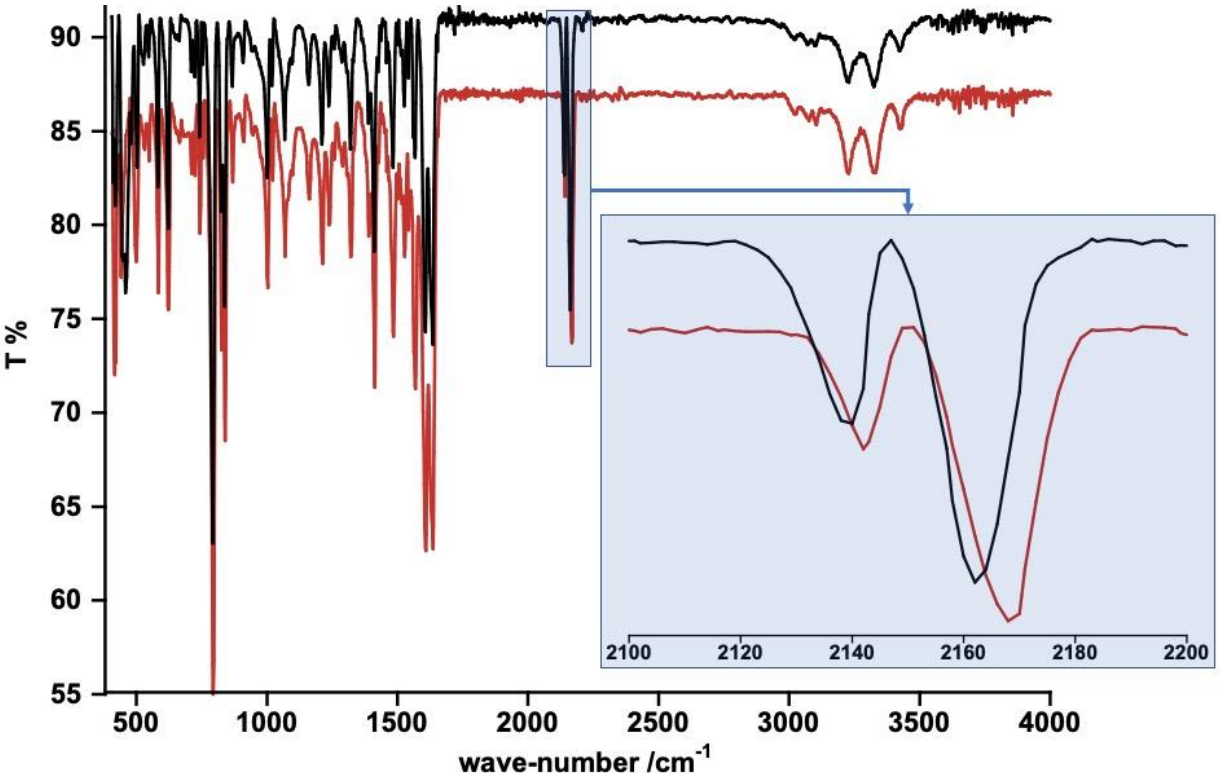

Table S1. Crystal data for **dpyanM** (M = Pd, Pt).

	**dpyanPd**		**dpyanPt**	
Empirical formula	$C_{40}H_{28}N_{14}Pd_2Fe$		$C_{40}H_{28}N_{14}Pt_2Fe$	
*M*r	973.41		1150.79	
Crystal system	monoclinic		monoclinic	
**T (K)**	**120**	**230**	**120**	**230**
Space group	*Pn*	$P2_1/n$	*Pn*	$P2_1/n$
a (Å)	10.3482(2)	10.4699(4)	10.3331(2)	10.5387(3)
b (Å)	14.2651(2)	13.4013(6)	14.3472(2)	13.2045(5)
c (Å)	13.7781(4)	14.9020(6)	13.6750(2)	14.8914(4)
β (°)	109.309(3)	104.836(4)	109.665(2)	104.138(3)
V (Å^3)	1919.49(8)	2021.20(15)	1909.09(6)	2009.49(11)
Z	2	2	2	2
D_c (mg cm^{-3})	1.684	1.599	2.002	1.902
F(000)	968	968	1096	1096
μ (Mo-K$_\alpha$) (mm^{-1})	1.351	1.283	7.736	7.349
Crystal size (mm)	0.03x0.15x0.15		0.02x0.10x0.10	
No. of total reflections	7797	4208	7645	4366
No. of reflections [$I>2\sigma(I)$]	6448	2813	7013	3465
R [$I>2\sigma(I)$]	0.0411	0.0499	0.0339	0.0404
wR [$I>2\sigma(I)$]	0.0824	0.1083	0.0649	0.0940
S	1.065	1.058	1.149	1.071

$R = \Sigma \,||Fo| - |Fc|| \,/\, \Sigma\, |Fo|$; $wR = [\,\Sigma\, [w(Fo^2 - Fc^2)^2] \,/\, \Sigma\, [w(Fo^2)^2]]^{1/2}$.
$w = 1/\,[\sigma^2(Fo^2) + (m\,P)^2 + n\,P]$ where $P = (Fo^2 + 2Fc^2)\,/\,3$;
m = 0.0271 (**1**), 0.0435 (**2**), 0.0219 (**3**), and 0.0485 (**4**);
n = 4.0407 (**1**), 5.5143 (**2**), 3.0495 (**3**), and 10.0038 (**4**)

Table S2. Relative angles between the different aromatic rings for **dpyanM** (M = Pd, Pt). See atom numbering in Figure 3.

Aromatic Ring A/Ring B	**dpyanPt**		Aromatic Ring A/Ring B	**dpyanPt**	**dpyanPd**
	Angle(°) **(120 K)**	Angle(°) **(120 K)**		Angle(°) **(230 K)**	Angle(°) **(230 K)**
Py(N12)/PhN(14)H_2	45.56	47.38	Py(N1)/PhN(4)H_2	43.77	46.23
PhN(14)H_2/Py(N13H13)$^+$	30.54	28.05	PhN(4)H_2/Py(N3H3)$^+$	8.19	7.06
Py(N12)/Py(N13H13)$^+$	76.06	75.39	Py(N1)/Py(N3H3)$^+$	37.82	40.83
Py(N1)/PhN(11)H_2	36.70	37.18			
PhN(11)H_2/ Py(N10H10)$^+$	27.46	25.71			
Py(N1)/Py(N10H10)$^+$	63.17	61.97			
Py(N1)/Py(N12)	78.51	78.86	Py(N1)/ Py(N1)	0	0

Figure S3. Short intermolecular contacts at 120 K for **dpyanM** (M = Pd, Pt) (marked in red correspond to those which are smaller than the sum of the van der Waals radii). Only interactions between layers n+1 and n are shown (red-blue rods). An equivalent distribution of short contacts occurs between layers n and n-1.

120 K		
NC···C(Ring)	dpyanPd d(Å)	dpyanPd d(Å)
C2···C24	3.362	3.334
C3···C16	3.361	3.350
C2···C32	3.507	3.471
C8···C37	3.241	3.270
C8···C38	3.305	3.308
C4···C16	3.589	3.578
C5···C20	3.585	3.591
C8···C39	3.616	3.578
C8···C40	3.651	3.578
C3···C24	3.572	3.554
C5···C31	3.579	3.616
C5···C24	3.653	3.586

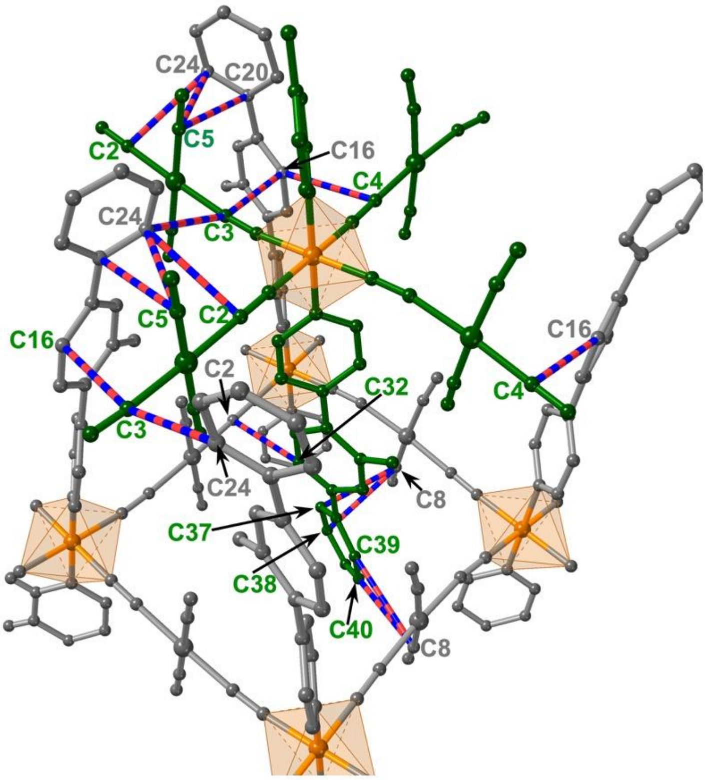

Figure S4. Short intermolecular π-π contacts at 120 K for **dpyanM** (M = Pd, Pt) (marked in red correspond to those close to the sum of the van der Waals radii).

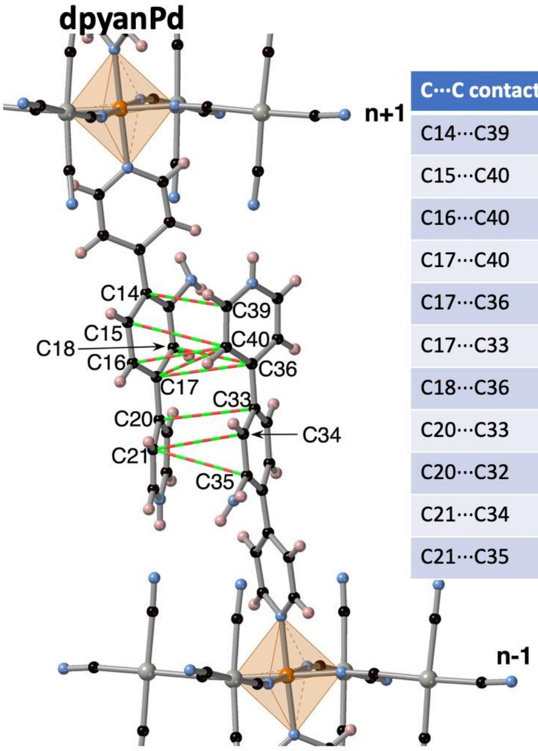

120 K

C···C contacts	dpyanPd	dpyanPt
C14···C39	3.467	3.456
C15···C40	3.566	3.522
C16···C40	3.432	3.433
C17···C40	3.485	3.507
C17···C36	3.551	3.558
C17···C33	-	3.598
C18···C36	3.552	3.512
C20···C33	3.595	3.530
C20···C32	-	3.498
C21···C34	3.481	3.495
C21···C35	3.557	3.534

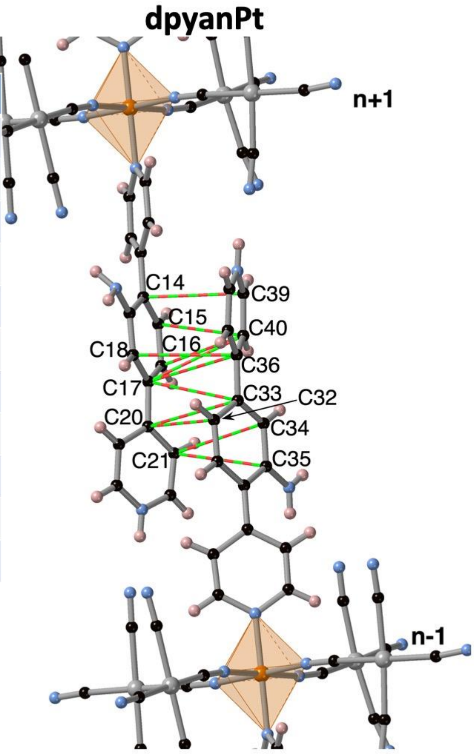

Supplement: Supplementary file 1 — ic3c01332_si_001.pdf [file ic3c01332_si_001.pdf]
